# Occult Kidney Dysfunction in Children With Transfusion-Dependent Thalassemia

**DOI:** 10.3389/fped.2021.754813

**Published:** 2021-11-22

**Authors:** Nurwahida Mohd Zikre, Nor A. Muhamad, Caroline S. Y. Eng, Nur E. Zailanalhuddin, Charles D. Lai, Jen C. Foo, Suet L. Yap, Hany Ariffin, Karmila Abu Bakar

**Affiliations:** ^1^Paediatric Unit, Faculty of Medicine, University Malaya, Kuala Lumpur, Malaysia; ^2^Sector for Evidence-Based Healthcare, National Institutes of Health, Ministry of Health, Kuala Lumpur, Malaysia; ^3^Paediatric Unit, Hospital Tuanku Ja'afar, Seremban, Malaysia

**Keywords:** thalassemia, nephropathy, transfusion-dependent, ferritin, iron chelator

## Abstract

**Background:** Thalassemia is the commonest hemoglobinopathy in Southeast Asia. Kidney dysfunction is an underreported sequelae in children with thalassemia. We conducted a retrospective study to identify the prevalence of and predisposing factors for kidney dysfunction in children with transfusion-dependent thalassemia (TDT).

**Method:** Abnormal kidney function was defined as children with a glomerular filtration rate (GFR) of <90 ml/min/1.73 m^2^ or a decline in GFR of >20 ml/min/1.73 m^2^ or presence of nephrotic range proteinuria within 3 years of commencing regular (every ≤6 weeks) red cell transfusion. Data analyzed were age at diagnosis of thalassemia, number of transfusion-years, iron chelation therapy, serum ferritin, and pre-transfusion hemoglobin levels.

**Results:** Eighty-one children were studied. Mean age was 11.72 ± 5.275 years. Thirty out of 81 (37%) demonstrated abnormal kidney function. Evidence of glomerular hyperfiltration was seen in 29/81 patients (25.85%) at their last clinic visit. This fraction was doubled [48/81 (59.3%)] when the cohort was tracked back by 3 years from the last clinic encounter. Age at diagnosis (RR, 1.157; 95% CI, 1.014–1.319; *p* = 0.03) and duration of receiving transfusions (RR, 0.984; 95% CI, 0.974–0.994; *p* = 0.001) were associated with increased risk of developing abnormal kidney function.

**Conclusion:** Abnormal kidney function in children with TDT may be overlooked by medical personnel without active screening measures. Children receiving regular red cell transfusions require systematic surveillance to enable early detection of kidney dysfunction and timely implementation of appropriate therapeutic interventions.

## Introduction

Thalassemia is the commonest inherited hemoglobinopathy, causing premature rupture of the red blood cells and ineffective erythropoiesis. It is estimated that about 8,000 people in Malaysia are living with thalassemia, with more than half (57%) of them dependent on regular blood transfusions (1).

In the absence of hematopoietic stem cell transplantation, red cell transfusion remains the mainstay of treatment in patients with transfusion-dependent thalassemia (TDT). Children with TDT are at risk of transfusion-related complications such as blood-borne infections, allergic reactions, acute lung injury, and circulatory overload (2), as well as end organ damage due to cumulative iron deposition (3–6). Iron deposition leads to damages likely explained by oxidative stress injury (7).

The thalassemic state affects the kidney in many ways. It is not till recent years that more evidence has emerged to link kidney dysfunction with thalassaemia (8–11). Ali et al. (12) examined 100 patients with beta-thalassemia major, comparing them to healthy controls. The mean age of their study population was 10 years old. They found that patients with thalassemia had a higher level of cystatin C, lower GFR, and higher urine albumin-to-creatinine ratio. There was a significant number of thalassemia patients in that cohort with kidney dysfunction despite being only in the first decade of life. Any persistent abnormality in the urine, abnormal structure, or GFR of <60 ml/1.73 m^2^/min is defined as chronic kidney disease. It is pivotal to detect the presence of abnormal kidney function early to prevent disease progression. Undoubtedly, chronic kidney disease in children carries a higher risk of accelerated cardiovascular disease and death (13).

Abnormal kidney function is expected to be seen in children with TDT as they enter adulthood, as a consequence of chronic transfusion. Lai et al. (14) conducted a similar study in an adult cohort of 81 patients with TDT who were regularly followed up for 10 years. They found that 15/81 (18%) patients had slow, but progressive decline in their eGFR. Those patients had a lower baseline eGFR, which could reflect those with preexisting kidney injury.

Various factors lead to kidney dysfunction in TDT, resulting from a combination of adversities of the chronic anemic milieu itself, iron overload, and exposure to certain types of iron chelation therapy. Chronic anemia promotes hypoxic injury to the tubulointerstitium and peritubular capillaries. With time, apoptosis, and mesenchymal epithelium transdifferentiation contribute to the gradual loss of normal kidney function (15). There are three iron chelators available for the management of iron overload in TDT: parenteral deferoxamine mesylate (Desferal^TM^, Novartis, Basel, Switzerland) and the oral agents deferiprone (Ferriprox^TM^, Apotex; Ontario, Canada) and deferasirox (Exjade^TM^, Novartis, Basel, Switzerland) (16). Much has been hypothesized on chelation therapy causing kidney dysfunction; from direct nephrotoxicity to alteration of intraglomerular hemodynamics following rapid depletion of iron from chelation (17, 18). However, not much is known about the prevalence of kidney dysfunction and its relation to the type of chelation therapy applied to these affected thalassemic children. To address these questions, we conducted a retrospective cohort study to measure prevalence and clinical characteristics of kidney dysfunction in children with TDT.

## Patients and Methods

### Study Population

This was a retrospective cohort study involving two tertiary-level medical institutions in Malaysia. Case note reviews were carried out by members of the clinical team. Patients aged 18 years or younger who had regular red cell transfusions (defined as intervals between transfusions of <6 weeks to maintain a hemoglobin level of >90 g/L for 1 year or more) at either University Malaya Medical Center or Hospital Tuanku Ja'afar between January 2020 to December 2020 were included. We excluded patients with thalassemia who only required periodic red cell transfusions (clinically managed as thalassemia intermedia) or newly diagnosed patients with TDT who received <1 year of regular transfusion.

### Data Collection

Patients' socio-demographic information (gender, age, and ethnicity), anthropometric measurements, blood pressure during blood transfusion, age at diagnosis, months of transfusion, pre-transfusion hemoglobin level, serum ferritin, serum creatinine, urine protein creatinine index, and type of iron chelation utilized were the variables recorded. These parameters were part of standard of care in the management of children with TDT. We used a predefined standardized data collection sheet for extracting data from the medical records. Pre-transfusion hemoglobin and ferritin levels during the preceding 12 months were obtained.

Evaluation of glomerular filtration rate (GFR) is best performed using clearance of inulin, but this method is impractical in day-to-day clinical practice. At the bedside, serum creatinine measurement is a feasible tool (19). Estimated glomerular filtration rate (eGFR) can be calculated using the Modified Schwartz formula (20). We compared the eGFR at several time points: patient's latest encounter with the managing team, T_0_, and at 3 years prior to that, T_−3_. The average of serum creatinine over a period of 12 months was used to calculate the eGFR. In this study, we defined glomerular hyperfiltration as eGFR of >140 ml/1.73 m^2^/min (21). Patients were classified to have abnormal kidney function if they develop any of the stated criteria: decline in eGFR of >20 ml/1.73 m^2^/min between T_−3_ and T_0_, eGFR of <90 ml/1.73 m^2^/min at T_0_, or presence of nephrotic range proteinuria (urine protein creatinine index >200 mg/mmol).

### Statistical Analysis

Descriptive statistics was performed to describe basic characteristics of the children with kidney dysfunction. We expressed numerical data as means with standard deviations. Categorical variables were expressed as numbers and percentages. Children with kidney dysfunction were compared to those without kidney dysfunction using chi-square tests for categorical variables and independent *t*-tests for continuous variables. A multivariate model analysis using logistic regression was performed to determine the predictors of kidney dysfunction. Data were analyzed by using the Statistical Package for Social Science software version 23.0 (SPSS Inc., Armonk, NY: IBM Corp).

## Results

**Figure 1:**
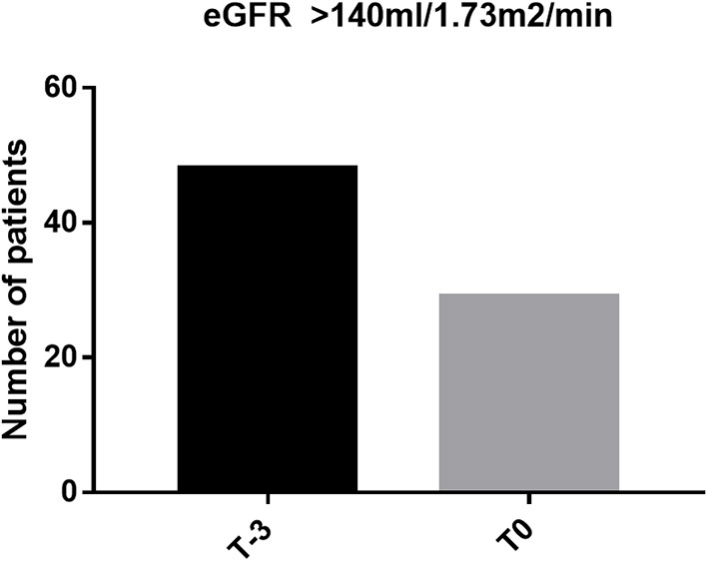
Proportion of glomerular hyperfiltration at last clinic visit (T_0_) and at 3 years before the last encounter (T_−3_).

Characteristics of the 81 patients are presented in Table 1. Forty-eight out of 81 patients (59.3%) were female. The majority of patients were of Malay ancestry (*n* = 62; 76.5%) while the remainder were of Chinese descent. Mean age in the study population was 11.72 ± 5.275 years old (2–22 years) and mean age at commencement of regular blood transfusion was 4.79 ± 3.693 years old. Thirty out of 81 (37%) patients fulfilled at least one of the criteria that defines abnormal kidney function: 1/81 had nephrotic range proteinuria (3.33%), 28/81 (34.6%) patients had declined in eGFR of more than 20 ml/min/1.73 m^2^ and 6/30 patients had established low eGFR of <90 ml/min/1.73 m^2^ (7.4%). Five patients had more than one abnormal feature. Evidence of glomerular hyperfiltration was seen in 29/81 patients (25.85%) at their last clinic visit (T_0_) (Figure 1). This proportion doubled (48/81, 59.3%) when the cohort was tracked back to 3 years from the last clinic encounter (T_−3_).

**Table 1:** Basic characteristics among normal and abnormal kidney function patients.

		**Kidney function**		***p*-value**
		**Normal *n* = 51**	**Abnormal *n* = 30**	
Age (years)		11.83 ± 5.390	11.54 ± 5.158	1.000
Age at diagnosis (years)		4.31 ± 3.761	5.62 ±3.480	0.432
Duration of transfusion (months)		89.25 ± 49.783	65.97 ± 36.676	0.014
eGFR at T_0(_ml/min/1.73m^2)^		146.97 ± 43.133	114.08 ± 25.372	1.000
eGFR at T_−3_ (ml/min/1.73m^2^)		136.67 ± 27.329	158.18 ± 34.722	1.000
Average transfusion (mL/kg/year)		177.55 ± 65.520	182.42 ± 46.068	1.000
Gender	Male	18 (35.3)	15 (50.0)	
	Female	33 (64.7)	15 (50.0)	<0.001
Race	Malay	36 (70.6)	26 (86.7)	
	Chinese	15 (29.4)	4 (13.3)	0.096
Height		132.60 ± 21.705	134.97 ± 22.461	1.000
SBP z-score		0.05 ± 0.959	0.09 ± 1.060	0.988
DBP z-score		0.09 ± 0.847	0.16 ± 1.076	0.98
Pre HB (g/dL) group	<8.5	11 (21.6)	6 (20.0)	
	Above 8.5	40 (78.4)	24 (80.0)	<0.001
Ferritin (μg/L)		2320.89 ± 1447.801	2154.86 ± 1061.055	1.000
Chelation therapy	Desferal	16 (31.4)	9 (30.0)	
	Deferasirox	19 (37.3)	12(40.0)	
	Deferiprone	4 (7.8)	1 (3.3)	<0.001
	Desferal + deferiprone	5 (9.8)	5 (16.7)	
	Desferal + deferasirox	6 (11.8)	2 (6.7)	
	None	1 (1.9)	1 (3.3)	

*Values are presented as mean ± standard deviation or number (%).GFR, glomerular filtration rate; SBP, systolic blood pressure; DBP, diastolic blood pressure; HB, hemoglobin*.

Urine protein creatinine index (UPCI) in one patient demonstrated nephrotic range proteinuria, while in others, it was <50 mg/mmol. This patient received deferasirox for 11 years. She had only proteinuria in the nephrotic range without overt nephrotic syndrome and her serum creatinine remained within normal limits. She had a renal biopsy but unfortunately that was suboptimal for diagnosis. After substituting her iron chelation therapy to desferioxamine, her urine protein gradually normalized.

**Figure 2:**
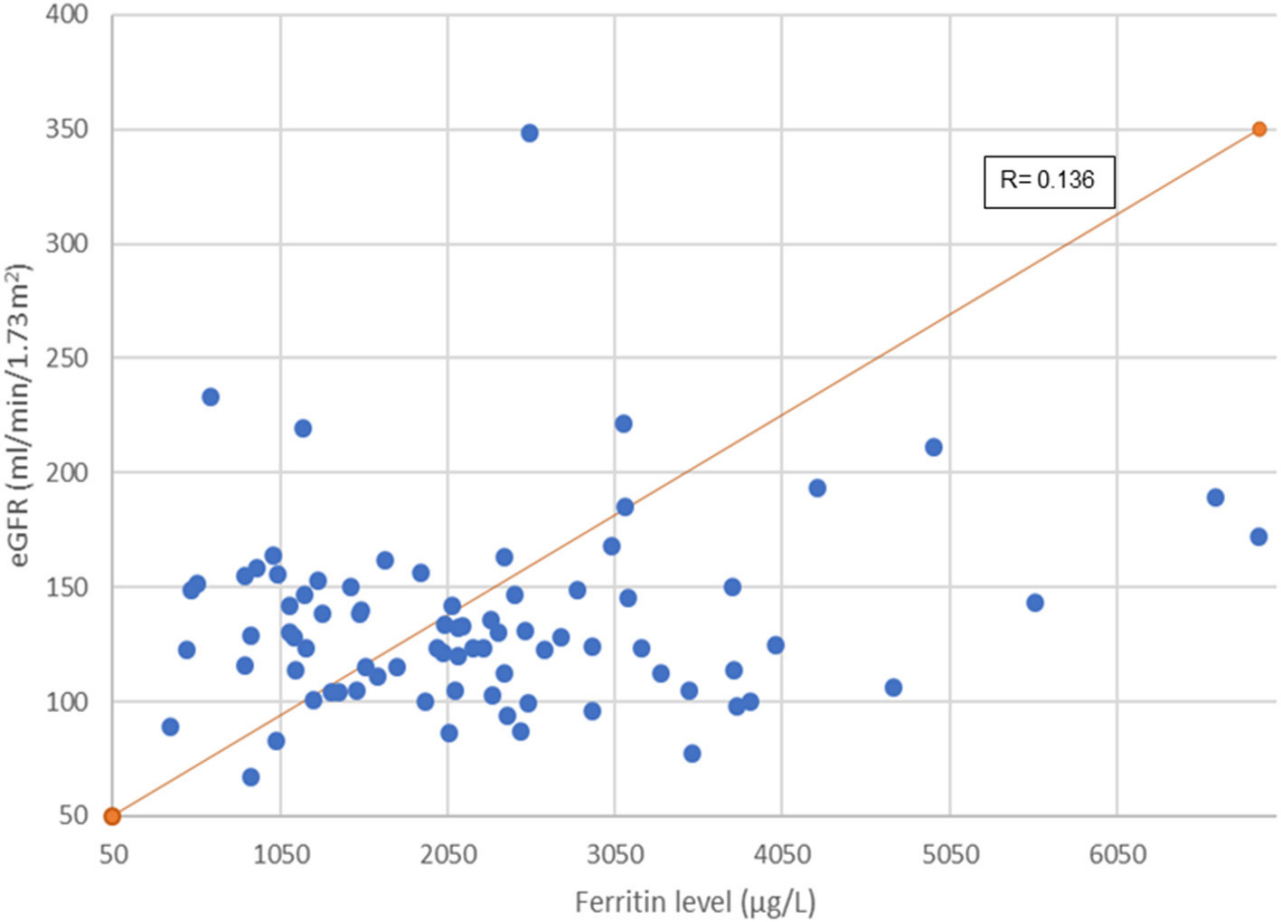
Scatter plot showing correlation of ferritin level and eGFR.

Figure 2 illustrates association between serum ferritin measurements and corresponding eGFR. Mean ferritin in the group with and without kidney dysfunction in our cohort did not show significant difference. However, it is interesting to note that 45 out of 81 (55.6%) patients had a very high serum ferritin level of >2,000 μg/L (range: 493–6,898 μg/L). Of this, a third (33.3%, 15/45) had glomerular hyperfiltration. A longer duration of transfusion (months) and younger age of transfusion commencement increased the risk of developing abnormal kidney function (Table 2).

**Table 2:** Multivariable analysis of patients with transfusion-dependent thalassemia.

		**B**	***df***	***p*-value**	**Adjusted relative risk (RR)**	**95% C.I.**	
						**Lower**	**Upper**
Age at diagnosis (years)		0.146	1	**0.030**	1.157	1.014	1.319
Duration of transfusion (months)		−0.016	1	**0.001**	0.984	0.974	0.994
Race	Malay		–				
	Chinese	0.378	1	0.346	1.459	0.664	3.206
Pre Hb (g/dL) group	<8.5		–				
	Above 8.5	−0.219	1	0.727	0.804	0.236	2.739
Chelation therapy	Desferal		5	0.411			
	Deferasirox	−0.817	1	0.424	0.442	0.060	3.271
	Deferiprone	−0.998	1	0.265	0.369	0.064	2.128
	Desferal + deferiprone	−2.422	1	0.140	0.089	0.004	2.212
	Desferal + deferasirox	0.355	1	0.769	1.427	0.134	15.244
	None	−0.830	1	0.494	0.436	0.040	4.707

*Significant when p < 0.05. The bold values mean they are statistically significant*.

## Discussion

We analyzed kidney function in patients with TDT and found that more than a third of our study subjects had evidence of dysfunction. Multivariate analysis showed that duration of exposure to transfusion is associated with the development of abnormal kidney function in children with thalassemia. This observation concurred with evidence in literature (12).

The mechanisms of transfusion-related renal dysfunction in patients with TDT have been reported by various authors. Chronic iron overload and anemia, which often coexist, are postulated to cause glomerular injury and tubular dysfunction. Zhou et al. (22) studied rats and Landing et al. (23) looking at autopsied patients; both reported glomerular and tubular changes of hemosiderosis. There were significant glomerulosclerosis, tubular atrophy, and interstitial fibrosis seen in those rats. The latter found visceral and parietal glomerular epithelial cells to be cellular, with increased mesangial matrix and hemosiderin deposits; greater in proximal and distal convoluted tubules. The histopathological changes are likely to be due to excess free iron, which stimulates the production of reactive oxygen species leading to cellular injury (7, 24).

It has been hypothesized that the underlying mechanism of iron chelators causing kidney injury is not related to a nephrotoxic effect of the drug *per se* but to “over-chelation,” leading to a relative depletion of iron and reduction in GFR (25). This hypothesis arose from observations of a high rate of serum creatinine increment in the desferoxamine arm (14% in patients with beta-thalassemia major and 22% in those with sickle cell disease) in published randomized trials (18, 26). Additionally, increases in serum creatinine were more often seen in patients who had a dramatic reduction of liver iron concentration and serum ferritin level as well as in subjects with lower transfusion rate and baseline iron indices (26). Causal mechanisms of GFR reduction with relative iron depletion include damage to mitochondrial function in tubular cells and production of adenosine and adenosine triphosphate (leading to activation of the tubuloglomerular feedback, vasoconstriction of the afferent preglomerular arterioles, and consequent reduction of GFR) and interference with the arachidonic acid cascade and the final production of prostaglandins (leading to an imbalance between vasodilating and vasoconstrictive prostaglandins), with consequent changes in intrarenal hemodynamics and GFR (25). Among the three iron chelators, deferasirox has reportedly more adverse events related to kidney such as increased serum creatinine, proteinuria, and, rarely, kidney failure (27). Desferioxamine-induced nephrotoxicity, however, though less common, involves mainly the tubular function (28–30). Prescribing iron chelators needs to be individualized, taking into consideration potential risks of progression of kidney injury in patients with preexisting kidney disease.

The role of chronic anemia in causing renal damage was explained by Nangaku (15). Tubulointerstitial damage, loss of peritubular capillaries, and interstitial fibrosis resulting from hypoxia will result in apoptosis or epithelial–mesenchymal transdifferentiation. The vicious cycle of renal damage continues, whose end point is end-stage renal disease (ESRD). We did not find anemia as a significant risk factor with our mean pretransfusion hemoglobin of 8.95 (±0.77) g/L. Hypertransfusion regime could be a protective factor, not only to prevent extramedullary hemopoiesis but also to suppress the cascade of reactive kidney injury.

Kidney dysfunction has been described in another common hemoglobinopathy, sickle cell disease (SCD). Ephraim et al. (31) described their cohort of 194 patients from as young as 5 years old with SCD. Chronic kidney disease was found in 39.2% of their participants, and across the CKD stages, they demonstrated an increasing trend in age. About two-thirds (68.8%) of their pediatric population showed glomerular hyperfiltration, while a third (31.2%) of the adults showed similar phenomenon. Observing this phenomenon even before they enter adulthood is worrisome.

Findings from our cohort reflect the model of glomerular hyperfiltration described by Brenner et al. (32). The proportion with glomerular hyperfiltration, in fact, doubled when our cohort was tracked back by 3 years. Maladaptation within the glomerular hemodynamics leads to hyperfiltration and progressive albumin excretion in urine. With time, this population shows “normal” GFR, which in real fact is an expression of the hyperfiltration state declining in renal function before they developed established kidney dysfunction (hypofiltration). There have been many opinions as to the mechanism that drives glomerular hyperfiltration in hemoglobinopathy. Notably, chronic anemia and previous splenectomy among individuals with hemoglobinopathy have been postulated to increase renal blood flow and alter intrarenal hemodynamics (11, 33). In our cohort, 33.3% of our patients with ferritin >2,000 μg/L showed evidence of hyperfiltration. Although these variables did not reach any statistical significance, such clinical observation is worth further exploration. Effect of iron deposition on the internal milieu within the glomerular is a potential research interest. There is much to be known on whether increased iron deposition in the myocardium indirectly affects the renal blood flow or whether elevated labile plasma iron in hyperferritinemic state triggers a cascade of oxidative stress injury to the glomeruli (34).

Children with chronic kidney impairment are usually growth retarded and hypertensive. Examining the *z*-score of height and blood pressure, we were not able to demonstrate a significant association of either parameter in our patients with abnormal kidney function. This suggests that in routine practice, standard parameters like blood pressure and anthropometric measurements are not adequately sensitive to detect subclinical kidney disease.

We acknowledge the limitations within our study. By default, our study was retrospective in nature. Our number of patients was small and requires further validation from other pediatric centers. Analysis of tubular function was also not possible in this study.

In conclusion, occult development of kidney dysfunction is seen early in children with TDT patients. Normal creatinine, growth, and blood pressure do not exclude the existence of kidney dysfunction in this group of patients. Careful interpretation of day-to-day clinical assessment will help clinicians to detect patients at risk. The role of kidney-specific biomarkers (cystatin-C and urine albumin excretion) and the potential effects of the renin–angiotensin–aldosterone system inhibition when hyperfiltration occurs are potential areas worth exploring. Revisiting the threshold to initiate chelation therapy may be necessary to prevent glomerular hyperfiltration, which may be overlooked by a falsely reassuring serum creatinine level.

## Data Availability Statement

The raw data supporting the conclusions of this article will be made available by the authors, without undue reservation.

## Ethics Statement

The studies involving human participants were reviewed and approved by National Medical Ethics Committee (NMRR-20-1849-55951) and Medical Research Ethics Committee at the University Malaya Medical Centre (UMMC) (MREC UMMC ID 2020721-8903). Written informed consent to participate in this study was provided by the participants' legal guardian/next of kin.

## Author Contributions

KA contributed to the conception and design of the study. NAM, NMZ, CL, SY, NZ, and JF organized the database. NAM performed the statistical analysis. KA, NAM, CE, and HA wrote the first draft and sections of the manuscript. All authors contributed to manuscript revision, read, and approved the submitted version.

## Conflict of Interest

The authors declare that the research was conducted in the absence of any commercial or financial relationships that could be construed as a potential conflict of interest.

## Publisher's Note

All claims expressed in this article are solely those of the authors and do not necessarily represent those of their affiliated organizations, or those of the publisher, the editors and the reviewers. Any product that may be evaluated in this article, or claim that may be made by its manufacturer, is not guaranteed or endorsed by the publisher.
